# Comparing Social Media and In-Person Recruitment: Lessons Learned From Recruiting Substance-Using, Sexual and Gender Minority Adolescents and Young Adults for a Randomized Control Trial

**DOI:** 10.2196/31657

**Published:** 2021-12-01

**Authors:** Jayelin N Parker, Alexis S Hunter, Jose A Bauermeister, Erin E Bonar, Adam Carrico, Rob Stephenson

**Affiliations:** 1 Center for Sexuality and Health Disparities University of Michigan Ann Arbor, MI United States; 2 Program on Sexuality, Technology & Action Research University of Pennsylvania Philadelphia, PA United States; 3 Department of Psychiatry University of Michigan Addiction Center University of Michigan Ann Arbor, MI United States; 4 Division of Prevention Science and Community Health University of Miami Miami, FL United States

**Keywords:** HIV testing, substance use, recruitment, sexual and gender minorities, youth

## Abstract

**Background:**

Recruiting large samples of diverse sexual and gender minority adolescent and young adults (AYAs) into HIV intervention research is critical to the development and later dissemination of interventions that address the risk factors for HIV transmission among substance-using, sexual and gender minority AYAs.

**Objective:**

This paper aimed to describe the characteristics of the samples recruited via social media and in-person methods and makes recommendations for strategies to recruit substance-using, sexual and gender minority AYAs, a hardly reached population that is a priority for HIV prevention research.

**Methods:**

Using data from a randomized control trial of an HIV and substance use intervention with sexual and gender minority AYAs, aged 15 to 29 years in southeastern Michigan (n=414), we examined demographic and behavioral characteristics associated with successful recruitment from a range of virtual and physical venues.

**Results:**

We found that paid advertisements on Facebook, Instagram, and Grindr offered the largest quantity of eligible participants willing to enroll in the trial. Instagram offered the largest proportion of transgender masculine participants, and Grindr offered the largest proportion of Black/African American individuals. Although we attempted venue-based recruitment at clubs, bars, community centers, and AIDS service organizations, we found it to be unsuccessful for this specific hardly reached population. Social media and geobased dating applications offered the largest pool of eligible participants.

**Conclusions:**

Understanding factors associated with successful recruitment has the potential to inform effective and efficient strategies for HIV prevention research with substance-using, sexual and gender AYAs.

**Trial Registration:**

ClinicalTrials.gov NCT02945436; https://clinicaltrials.gov/ct2/show/NCT02945436

**International Registered Report Identifier (IRRID):**

RR2-10.2196/resprot.9414

## Introduction

Hardly reached populations refer to groups that are traditionally under-recruited into health research. Such populations were, until recently, referred to as hard-to-reach, but recent literature has redefined them as *hardly reached*, switching the emphasis to researchers’ inability to recruit rather than the population’s inability to be recruited [1]. Hardly reached populations often experience high levels of structural vulnerability (ie, homelessness or incarceration), creating significant barriers to their participation in research. These hardly reached groups may also experience high levels of stigma and discrimination associated with their identity (ie, transphobia or homophobia) or behaviors (ie, people who use substances) that act as a barrier to their research in participation [2,3]. An alternative term, *hidden population*, is often used to refer to those who may not wish to be found or contacted (eg, people who use substances or undocumented migrants) [4]. For example, people who use or misuse substances may be reluctant to disclose behaviors that would make them eligible for research participation due to fears of illegal behaviors being reported to authorities or the high level of stigma associated with this behavior [5-7].

While hardly reached populations often include groups with minority identities (ie, racial and ethnic minorities or sexual and gender minorities) [4,8,9], they may also include other populations that are under-recruited due to geographical location (ie, rural populations), lack of access to health services (for the recruitment of clinic-based populations), or access to technology (for recruitment of online samples) [1,3,4]. Difficulties experienced in engaging hardly reached populations in research studies may be particularly heightened when individuals live with the intersectionality of being a sexual or gender minority and engaging in a stigmatized behavior of substance use. The stigma associated with underage drinking or use of illegal drugs could significantly decrease the likelihood of participation in research [2]. In HIV research, the potential under-recruitment of substance-using young men who have sex with men (YMSM) is particularly problematic given data illustrating the growing incidence of HIV and striking associations between substance use and HIV risk [10]. Sexual and gender minority youth are increasingly important to recruit into prevention research to accurately develop prevention strategies that represent and are tailored toward diverse communities. Strategies are needed to recruit youth that represent local demographics and geography (ie, in states such as Michigan with large rural populations) and risk groups in localized epidemics [11].

Venue-based recruitment is a modified venue-time-spaced screening approach implemented by behavioral surveillance and involves listing physical venues where target populations can be found and using this list to identify times to recruit potential participants. Venue-based recruitment has served as a viable way to connect with substance-using, sexual and gender minority, adolescent and young adults (AYAs) and can occur through locations such as bars and clubs, AIDS service organizations, or street outreach by research study staff. Multiple studies have demonstrated that recruitment through venues such as bars or nightclubs can often yield high numbers of substance-using, sexual and gender minority AYAs [12-14]. However, the age restrictions of bars and clubs poses a barrier to the recruitment of YMSM under the age of 21 years. This age requirement could possibly result in fewer adolescents recruited or additional effort required from research staff, leaving online-based recruitment as a more effective way to recruiting sexual and gender minority AYAs into HIV research [15].

To counteract the challenges of venue-based sampling and community-based outreach, researchers are increasingly using internet-based recruitment to recruit populations who may be hardly reached through traditional sampling methods [16]. Using the internet for social media–targeted recruitment has been reported as a feasible strategy for recruiting large samples of men who have sex with men (MSM) [16-19] as well as AYAs who engage in substance use [20,21]. Compared with traditional recruitment strategies, social media is attractive for its wide geographic reach, cost effectiveness, usability, and capacity for engaging hardly reached, isolated, or minority populations [22-24]. Social media (eg, Facebook, Instagram, and other socially oriented platforms such as Grindr) has been shown to be a cost-effective recruitment strategy [25-27] to recruit AYAs who have demographic profiles reflective of the general AYA population. Additionally, social media and internet-based recruitment efforts can reach rural areas, where opportunities for venue-based recruitment can be limited [28]. Based on a sample of 8252 participants, Christensen et al [29] found that social media recruitment was more efficient (total number of participants enrolled); had an average lower cost per recruited participant, compared with in-person methods; and was found to be cost-effective and rapid, with researchers paying, on average, US $17 per completer (range US $1.36–$110). Thornton et al [30] reported that 86% of studies reported similar representativeness between online and offline samples, with no systematic gender or age differences. The use of social media platforms can allow for a broader range of hardly reached YMSM populations (ie, rural areas) [31]. Facebook [32-34] and the dating application Grindr [18,35,36] have yielded successful recruitment of YMSM, though Grindr at a higher cost [24]. However, recruitment via the internet has the potential to exclude YMSM without access to the internet, which often includes individuals who are ethnic minorities or socioeconomically disadvantaged.

Here, we describe a case study of recruiting substance-using, young, sexual and gender minority AYAs (aged 15-29 years) in southeastern Michigan for a randomized controlled trial of a substance use and HIV prevention counseling intervention. This paper describes and contrasts 2 recruitment methods (in-person, venue-based recruitment and recruitment via the internet using social media targeted advertising), describes the characteristics of the samples recruited via each method, and adds recommendations to the literature for programs aiming to recruit substance-using YMSM, a hardly reached population that it is urgent to include in HIV prevention research.

## Methods

### Study Design

Project Swerve is a randomized control trial testing the efficacy of a substance use brief intervention for creating gains in HIV and sexually transmitted infection (STI) testing among young, substance-using, sexual and gender minority communities, aged 15 to 29 years. Full details of the intervention and research protocol can be found in [32].

### Eligibility

Eligibility criteria for the trial participants included self-reported (1) sex assigned male at birth and currently identifying as man/male, woman/female/trans feminine, and/or gender nonbinary or assigned female at birth and currently identifying as man/male/trans masculine; (2) age between 15 and 29 years at the time of screening; (3) negative or unknown HIV status at screening; (4) past 3-month drug use or binge drinking (eg, stimulants, hallucinogens, opioids, sedatives, amyl-nitrite, or club drugs with alcohol and/or cannabis); (5) condomless anal or oral sex with a self-identified man in the 6 months prior to enrollment; and (6) resident of southeastern Michigan at the time of screening.

### Recruitment

#### Overview

Recruitment of participants took place from April 2017 to September 2019 and consisted of 2 modes of recruitment: (1) online recruitment through social media and dating applications and (2) in-person recruitment at local venues. Online recruitment consisted of paid advertisements on social media platforms, including those aimed at general audiences—Facebook, Instagram, Snapchat, Reddit, and Google Ads—as well as networking mobile apps targeted toward sexual and gender minorities—Grindr, Scruff, Jack’d, and Bareback Realtime. Web-based recruitment also included unpaid advertisements through a health research portal based at the university, Tumblr, and Twitter. We used Completely Automated Public Turing test to tell Computers and Humans Apart (CAPTCHA) during the screening process to verify for fraud. Additionally, email addresses could only register once and were sent verification links to complete registration.

#### Online Recruitment

Online advertisement photos were purchased from stock photo websites and included people from a range of gender identities, races, and ethnicities. Language used in online advertisements included “Get paid to participate in an HIV testing program” and “Participate in a paid university study about health and HIV testing.” To supplement paid online static advertising, recruitment videos were also created through an online video creation platform that provides stock footage and images. These short, <15-second videos consisted of people holding hands and pride flags and used the same language as the static advertisements. These were posted as video advertisements on Facebook, Instagram, Reddit, Tumblr, and Twitter.

We ran 3 campaigns at a time on Facebook and Instagram: 2 for cisgender MSM and 1 for transgender individuals. Facebook and Instagram allow advertisers to target populations based on age, location, and interests, which allowed us to narrow our impressions to those between 15 and 29 years old, residing within southeastern Michigan, and within specific search interests (eg, gay pride, Gay Straight Alliance [GSA], gay, bisexual, transgender, gay bars). Facebook and Instagram offer an advertisement boost option, which costs US $20 and increases the advertisement’s reach for a limited amount of time.

Snapchat advertisements cost US $50 a day and could be targeted by interests (eg, lifestyle, sports, technology), location (eg, cities within our participant range), and demographics (eg, age, gender, language). We began Snapchat advertisements at the end of June 2018, running them every weekend through the end of July 2018 to see if they would bring in any new participants. After we found Snapchat ads to bring in some new eligible participants, we increased the frequency of our advertisements to 7 days every other week during August 2018 and increased once more to month-long advertisements from September to mid-November 2018. Snapchat did not bring in enough new eligible participants for the project, and we decided to end advertisements in November 2018.

Reddit advertisements were developed in November 2018 and ran through December 2018. After consideration of our project and its inclusion of minors, Reddit chose not to allow us to advertise our project through their platform, where recruiting only people aged ≥18 years old is acceptable. Our advertisements were reported by users 5 times in 2 months, and organic posts were deleted from threads by owners.

Grindr, a geosocial networking application, offered 2 different advertisements: banner (at the top of the screen) and interstitial (a full-page advertisement). We created an advertisement for each: a banner “Interested in getting paid to test for HIV/STIs? Swerve is a testing program looking for young gay men and Trans folks. Click here!” and a full-page, interstitial advertisement “Get paid to test for HIV/STIs by joining Swerve - A testing program for young gay men and Trans folks.” Grindr advertisements target MSM and can target specific cities, which helps to narrow down the impressions for people within the enrollment criteria. Grindr cannot target age. We ran 2 flat-rate advertisements: April to October 2017 and November 2017 to April 2018. In 2018, Grindr changed their advertisement approach to bids to be advertised on the platform. We ran this set of Grindr campaigns from August 2018 to August 2019, with a typical banner campaign running at US $1250 total, with a US $40 a day spending cap in place. A typical interstitial campaign with a capped budget cost US $1000 total, had a US $35 a day spending cap, and was auto-placed throughout the day.

Scruff, a networking application also targeting MSM, offered advertisements that cost a flat rate of a minimum US $500 for 2 weeks, with a cost per 1000 impressions option. At the time, Scruff’s advertisements were full page with a call-to-action button, which linked the advertisement directly to the landing page. Scruff can target by location and, similarly to Grindr, cannot target by age. We ran a full-page advertisement, the same as on Grindr, for 1 month and targeted Detroit, Michigan with a 50-mile radius. Jack’d, another geosocial networking application for the gay, bisexual, and transgender community, offered only banner ads for public health–based research projects when we were using their advertising platform from June 2018 to July 2018. The same banner advertisement used on Grindr was used on Jack’d and cost US $2000 for 1 month of advertisements.

We created a Twitter account in October 2017 and used it to boost our online presence. We had a pinned tweet with a link to the Swerve landing page and would tweet our recruitment materials (videos and photos), post when we were at a conference, and share articles related to sexual health. Our final post was in October 2019. Similarly, we created a Tumblr account to boost engagement and posted recruitment materials with tags (eg, pride, gay, Michigan, bisexual, transgender, mtf, ftm, HIV, HIVtesting, knowyourstatus, healthyliving, publichealth). We made a total of 10 posts to Tumblr between January 2018 and February 2019.

Bareback Realtime is a web service that MSM use to meet other men. They offer free Quick Connect Ads, which are typically used to meet other people. We made our advertisements last for 6 hours at a time and used language similar to that used with Grindr. Bareback Realtime was posted an average of once per week in varying cities in southeastern Michigan, and we ran the advertisements from May 2017 to September 2019. Bareback Realtime was not successful, and advertisements were placed sparingly throughout the recruitment period.

The university at which this trial is housed offers a health research portal for research staff to share their trials with the public. The name, description, study topic, participant involvement, compensation, location, inclusion and exclusion criteria, and additional screening questions were options for development of the research page. We used HIV, substance abuse, and sexual health as our study topics; explained what the project entailed; explained the breakdown of compensation per visit; listed Ypsilanti, Detroit, and Flint as study locations; listed age of 15-29 years old and “male identifying” as inclusion criteria; and did not include any additional screening questions. When people marked that they were interested in the study, we sent them a link to the landing page to participate in the eligibility screener.

#### Venue-Based Recruitment

Venue-based recruitment took place from October 2017 to September 2019. To reach our target population, potential participants were systematically sampled from a predeveloped list of public venues where sexual and gender minority AYAs were known to frequent, which included lesbian, gay, bisexual, transgender, queer, plus (LGBTQ+)–friendly bars, clubs, drag shows, restaurants, and cafés; events in Michigan (eg, Pride); health fairs and wellness centers (testing centers); campus-based events at high schools and colleges in southeastern Michigan; and social organizations (eg, local GSA chapters).

Staff went to at least 10 locations each week and always to 2 different locations on Friday and Saturday nights. Approximately 30 locations were potential venues for each month’s selection, where some venues were attended more than once per week. Some venues were not chosen to attend due to lack of permission from management, weather concerns for outdoor events, seasonal events, or lack of regular attendance by patrons. An average of 40 locations were attended each month.

All potential participants were approached within staffing and time limits. Staff were required to spend at least 2 hours recruiting during each scheduled shift, and if a venue had a low turnout, staff would move on to a second location to ensure at least 10 people were screened during each recruitment shift. Participants were offered to take the screener at the time of recruitment and if they refused, were handed a palm card (a small postcard-sized flyer with information regarding the trial). Palm cards and coasters were left at locations for people to pick up on days and at times when staff were not actively recruiting. We purchased or signed up for table space at local pride events, conferences, and health fairs at colleges and community centers. At these events, we passed out recruitment materials and approached potential participants to take the eligibility screener on tablets and left a sign-up sheet on the table for people to leave their name, phone number, email address, and age so we could contact them for screening if they decided not to take the eligibility screener on-site.

Staff were trained to approach potential participants by introducing themselves, the project, and their purpose of recruiting. T-shirts with the project logo were worn to show legitimacy of the project. Staff always recruited in groups of 2 or more after 6 pm to ensure safety, and groups of 4 or more would split up and attend events in different cities (ie, Detroit and Ann Arbor) to recruit more participants in each shift.

### Measures

#### Sociodemographic Characteristics

In the eligibility screening survey, we asked demographic questions on sexual orientation, gender identity, sex assigned at birth, age, race, ethnicity, zip code, and school enrollment status. Participants were asked to identify their gender, using the check all that apply method, with options being female, male, trans woman, trans man, gender queer/nonconforming, and other. Participants indicated their sexual orientation as straight or heterosexual, gay or homosexual, bisexual, same gender loving, queer, or other. Participants were not eligible if they stated their gender was female and their sexual orientation was bisexual, same gender loving, or queer or if their gender was male and sexual orientation was heterosexual. Participants indicated their race as White/Caucasian, Black/African American, Asian/Pacific Islander, Middle Eastern, or other, and ethnicity was identified by Hispanic/Latino or not. Participants were asked to check all that apply, allowing us to identify racial and ethnic subgroups (eg, Black Latino, Asian Latino).

#### HIV Status and Sexual Experience

We asked participants to indicate if they had ever tested positive for HIV, and those with self-reported unknown or presumed negative HIV status were eligible. We asked participants to identify if they had condomless oral or anal sex with someone who identifies as a man in the past 6 months at the time of screening.

#### Substance Use

We asked participants to identify any substances used in the last 3 months using the check all that apply method. Substances included tobacco or nicotine products, more than 5 standard alcoholic drinks in each day or night, cannabis, cocaine or crack, heroin, hallucinogens, inhalants, prescription pain relievers, prescription tranquilizers, prescription stimulants, prescription sedatives, methamphetamine, or none of the above. Participants were eligible if they reported using substances other than tobacco or alcohol and/or if they reported alcohol use at a rate of 5 or more standard drinks per day.

### Statistical Analysis

We conducted a descriptive analysis across all recruitment platforms to examine differences in demographic characteristics (ie, gender identity, race, age) and substance use. The second step of our analysis focused on eligibility and enrollment from each recruitment source, where total eligibility and enrollment rates were calculated for the study overall as well as each recruitment source. Models were run in Stata Statistical Software version 16 (StataCorp LLC, College Station, TX).

## Results

### Eligibility

We had a total of 17,328 visits to the eligibility screener on the landing page, with 6274 (36.2%) completing screeners. Of those who completed the screener, 623 (623/6274, 9.92%) consented and were eligible for the trial, and 580 (580/623, 93.10%) of those who were eligible responded with their source of recruitment in their eligibility screener (Table 1). This question was added to the eligibility screener after 43 participants had taken the screener.

**Table 1:** Participant eligibility and enrollment by recruitment source.

Recruitment source	Screened eligible (n=580), n (%)	Enrolled (n=217), n (%)	Enrollment rate (total=37.4%), %
BBRT	1 (0.2)	1 (0.5)	100
Facebook	244 (42.1)	85 (39.2)	34.8
Friend	31 (5.3)	12 (5.5)	38.7
Google Ad	2 (0.3)	1 (0.5)	50.0
Grindr	135 (23.2)	54 (24.9)	40.0
Instagram	63 (10.8)	24 (11.1)	38.1
Jack’d	3 (0.5)	1 (0.5)	33.3
Other	3 (0.5)	1 (0.5)	33.3
Reddit	4 (0.7)	3 (1.4)	75.0
Scruff	10 (1.7)	3 (1.4)	30.0
Snapchat	2 (0.3)	1 (0.5)	50.0
Tumblr	13 (2.2)	5 (2.3)	38.5
Twitter	2 (0.3)	1 (0.5)	50.0
University health research	42 (7.2)	20 (9.2)	47.6
Venue	23 (3.9)	6 (2.8)	26.1

### Recruitment

Of the 580 participants who reported their source of recruitment, Facebook advertisements offered the largest quantity of eligible participants (244/580, 42.1%) followed by advertisements on Grindr (135/580, 23.2%) and Instagram (63/580, 10.8%; Table 2). Other paid advertisements on the dating application Jack’d (3/580, 0.5%) yielded a low number of eligible participants. The health research portal website, an unpaid university level study participant portal, enrolled 42 (42/580, 7.2%) of eligible participants. A small number of eligible participants indicated a friend (31/580, 5.3%) or other (3/580, 0.5%) referred the eligible participants to the study for enrollment.

Unpaid social media advertisements including Google Ads (2/580, 0.51%), Snapchat (2/580, 0.3%), Reddit (4/580, 0.7%), Tumblr (13/580, 2.2%), Twitter (2/580, 0.3%), and the dating applications Scruff (10/580, 1.7%) and BBRT (1/580, 0.2%) offered the smallest quantities of eligible participants.

**Table 2:** Demographics of the eligible participants by recruitment source.

Characteristic		Total eligible sample, n (%)	Recruitment source^a^, n (%)														
			A	B	C	D	E	F	G	H	I	J	K	L	M	N	O
Sample size		580	1	244	31	3	135	63	3	3	4	10	2	13	2	42	23
**Gender identity**																	
	Female	2 (0.3)	0 (0)	2 (0.8)	0 (0)	0 (0)	0 (0)	0 (0)	0 (0)	0 (0)	0 (0)	0 (0)	0 (0)	0 (0)	0 (0)	0 (0)	0 (0)
	Male	475 (76.2)	1 (100)	185 (75.8)	27 (87.1)	4 (100)	121 (89.6)	51 (80.9)	2 (0.3)	2 (66.7)	0 (0)	10 (100)	2 (100)	9 (69.2)	2 (100)	40 (95.2)	19 (82.6)
	Trans woman/female		0 (0)		0 (0)	0 (0)		0 (0)	0 (0)	0 (0)	0 (0)	0 (0)	0 (0)	0 (0)		0 (0)	0 (0)
	Trans man/male	66 (10.6)	0 (0)	35 (14.3)	4 (12.9)	0 (0)	5 (3.7)	12 (19.1)	0 (0)	0 (0)	3 (75.0)	0 (0)	0 (0)	2 (15.4)	0 (0)	2 (4.8)	3 (13.0)
	Gender queer/nonconforming	16 (69.6)	0 (0)	6 (2.5)	0 (0)	0 (0)	6 (0.7)	0 (0)	1 (0.2)	1 (33.3)	1 (25.0)	0 (0)	0 (0)	0 (0)	0 (0)	0 (0)	1 (4.3)
	Other	1 (0.2)	0 (0)	0 (0)	0 (0)	0 (0)	0 (0)	0 (0)	0 (0)	0 (0)	0 (0)	0 (0)	0 (0)	1 (7.7)	0 (0)	0 (0)	0 (0)
**Race and ethnicity**																	
	White/Caucasian	352 (60.7)	1 (100)	155 (63.5)	18 (58.0)	2 (66.7)	69 (51.1)	38 (60.3)	0 (0)	1 (33.3)	4 (100)	7 (70.0)	1 (50.0)	6 (46.1)	1 (50.0)	29 (69.0)	19 (82.6)
	Black/African American	90 (15.5)	0 (0)	38 (15.6)	4 (12.9)	0 (0)	28 (10.7)	7 (11.1)	3 (100)	1 (33.3)	0 (0)	0 (0)	1 (50.0)	4 (30.8)	0 (0)	4 (9.5)	0 (0)
	Asian/Pacific Islander	20 (3.4)	0 (0)	7 (2.8)	0 (0)	0 (0)	5 (3.7)	6 (9.5)	0 (0)	0 (0)	0 (0)	0 (0)	0 (0)	0 (0)	0 (0)	2 (4.8)	0 (0)
	Middle Eastern	13 (2.2)	0 (0)	3 (1.2)	2 (6.4)	0 (0)	4 (2.9)	2 (3.2)	0 (0)	0 (0)	0 (0)	2 (20.0)	0 (0)	0 (0)	0 (0)	0 (0)	0 (0)
	Hispanic or Latinx	45 (7.8)	0 (0)	13 (5.3)	3 (9.7)	1 (33.)	17 (12.6)	7 (11.1)	0 (0)	0 (0)	0 (0)	0 (0)	0 (0)	1 (7.7)	0 (0)	1 (2.4)	2 (8.7)
	Native or American Indian	8 (1.4)	0 (0)	3 (1.2)	1 (3.2)	0 (0)	1 (0.7)	1 (1.6)	0 (0)	0 (0)	0 (0)	0 (0)	0 (0)	0 (0)	2 (15.4)	0 (0)	0 (0)
	Biracial	49 (8.4)	0 (0)	24 (9.8)	2 (6.4)	0 (0)	11 (8.1)	2 (3.2)	0 (0)	1 (33.3)	0 (0)	1 (10.0)	0 (0)	0 (0)	0 (0)	6 (14.3)	2 (8.7)
	Multiracial (≥3)	3 (0.5)	0 (0)	1 (0.4)	1 (3.2)	0 (0)	0 (0)	0 (0)	0 (0)	0 (0)	0 (0)	0 (0)	0 (0)	0 (0)	1 (50.0)	0 (0)	0 (0)
**Age (years)**																	
	15-19	120 (20.7)	0 (0)	43 (17.6)	6 (19.4)	1 (33.3)	29 (21.5)	23 (36.5)	2 (66.7)	0 (0)	3 (75.0)	0 (0)	1 (50.0)	3 (23.1)	0 (0)	4 (9.5)	7 (30.4)
	20-24	284 (48.9)	0 (0)	117 (47.9)	18 (58.1)	2 (66.7)	68 (50.4)	28 (44.4)	1 (33.3)	2 (66.7)	1 (25.0)	3 (30.0)	1 (50.0)	6 (46.2)	1 (50.0)	20 (47.6)	15 (65.2)
	25-29	176 (30.3)	1 (100)	84 (34.4)	7 (22.6)	0 (0)	38 (28.1)	12 (19.0)	0 (0)	1 (33.3)	0 (0)	7 (70.0)	0 (0)	4 (30.8)	1 (50.0)	18 (42.8)	1 (4.3)
**Substance use**																	
	Illicit substances with alcohol and/or marijuana (polysubstance use)	231 (39.8)	1 (100)	96 (39.3)	20 (32.3)	2 (66.7)	50 (37.0)	19 (30.1)	1 (33.3)	2 (66.7)	2 (50.0)	6 (60.0)	1 (50.0)	9 (69.2)	2 (100)	20 (47.6)	9 (39.1)
	Illicit substances only	15 (2.6)	0 (0)	9 (3.7)	0 (0)	0 (0)	2 (1.5)	4 (6.3)	0 (0)	0 (0)	0 (0)	0 (0)	0 (0)	0 (0)	0 (0)	0 (0)	0 (0)
	Alcohol and marijuana only	166 (28.6)	0 (0)	77 (31.6)	13 (41.9)	1 (33.3)	31 (22.9)	19 (30.1)	0 (0)	1 (33.3)	0 (0)	2 (20.0)	0 (0)	3 (23.1)	0 (0)	11 (26.1)	8 (34.7)
	Alcohol only	44 (7.6)	0 (0)	14 (5.7)	3 (9.7)	0 (0)	15 (11.1)	6 (9.5)	0 (0)	0 (0)	1 (25.0)	1 (10.0)	0 (0)	0 (0)	0 (0)	3 (7.1)	1 (4.3)
	Marijuana only	124 (21.4)	0 (0)	48 (19.7)	5 (2.0)	0 (0)	37 (27.4)	15 (23.8)	2 (66.7)	0 (0)	1 (25.0)	1 (10.0)	1 (50.0)	1 (7.7)	0 (0)	0 (0)	5 (21.7)

^a^A: BBRT; B: Facebook; C: Friend; D: Google Ad; E: Grindr; F: Instagram; G: Jack’d; H: Other; I: Reddit; J: Scruff; K: Snapchat; L: Tumblr; M: Twitter; N: University; O: Venue.

The screened eligible participant sample (n=580) was composed mostly of those who self-identified as male (475/580, 81.9%), followed by transgender men (66/580, 11.4%), and transgender women (20/580, 3.4%). Most identified as White/Caucasian (382/580, 65.8%), followed by Black/African American (90/580, 15.5%) and biracial (49/580, 8.4%). The largest proportion of participants who screened eligible were in the age range of 20-24 years (314/580, 54.1%). Facebook offered the largest volume of transgender women (16/244, 6.6% of those eligible on Facebook), where Grindr (3/135, 2.2%) and Tumblr (1/13, 8%) were the only other sources to recruit transgender women. Again, Facebook recruited the highest volume of transgender men (35/244, 14.3%), but friend referral (4/31, 13%), Grindr (5/135, 3.7%), Instagram (12/63, 19%), Reddit (3/4, 75%), Tumblr (2/13, 15%), and the university research portal (2/42, 5%) also recruited transgender men.

Of those recruited on Facebook, 155 (155/244, 63.5%) were White, and 38 (38/244, 15.6%) were Black/African American, compared with Grindr, where 69 (69/135, 51.1%) were White and 28 (28/135, 20.7%) were Black/African American, and Instagram, where 38 (38/63, 60.3%) were White and 7 (7/63, 11%) were Black/African American. Reddit recruited 100% (4/4) White participants, and the university research portal primarily recruited White participants (29/42, 69%). Reddit and Jack’d recruited the highest percentage of those aged 15-19 years (3/4, 75%; 2/3, 67%, respectively), although the volume was very low.

Of the 580 eligible participants, polysubstance use (eg, stimulants, hallucinogens, opioids, sedatives, amyl-nitrite, or club drugs) with alcohol or cannabis was the most commonly self-reported substance use behavior (231/580, 39.8%), followed by alcohol and cannabis use only (166/580, 28.6%) and cannabis use only (124/580, 21.4%). This pattern was relatively consistent across participants from all recruitment sources. Facebook and Grindr offered the largest proportions of polysubstance and alcohol users. The highest percentages of those reporting alcohol use only were on Instagram (15/63, 10%), via friend referral (3/31, 10%), and on Reddit (1/4, 25%, although this was only 1 participant), showing most participants were using more than alcohol. The use of cannabis only was most reported by those recruited through Jack’d (2/3, 67%), Reddit (1/4, 25%), Grindr (37/135, 27.4%), and venue-based sampling (5/23, 22%), although this only represents 7.8% (45/580) of the recruited participants.

Recruiting this hardly reached population was costly for the study, as it cost US $281.14 per participant to enroll in the study, and over US $61,000 of our budget to recruit (Table 3). Snapchat, Jack’d, and venue-based recruitment were the most expensive per enrolled participant, costing 26.4% ($16,131/$61,006.94) of the total recruitment cost and only enrolled 8 participants.

**Table 3:** Cost of participant recruitment by recruitment source.

Recruitment source	Total cose (US $)	Eligible participants		Enrolled participants	
		Number of participants	Cost per participant (US $)	Number of participants	Cost per participant (US $)
BBRT	0	1	0	1	0
Facebook and Instagram	16,904.58	307	55.06	109	155.09
Friend	0	31	0	12	0
Google Ad	226.70	3	75.56	1	226.70
Grindr	27,085.55	135	200.63	54	501.58
Jack’d	2000.00	3	666.67	1	2000.00
Other	0	3	0	1	0
Reddit	159.11	4	39.77	3	53.04
Scruff	500.00	10	50.00	3	166.67
Snapchat	5330.00	2	2665.00	1	5330.00
Tumblr	0	13	0	5	0
Twitter	0	2	0	1	0
University health research	0	42	0	20	0
Venue	8801.00	23	382.66	6	1466.83
Total	61,006.94	580	105.18	217	281.14

## Discussion

We learned several lessons from recruiting and enrolling young, substance-using, sexual and gender minority AYAs into a large, randomized control trial in southeastern Michigan. Online advertising across a variety of platforms led to more success in recruiting a large volume of diverse, young, substance-using, sexual and gender minority individuals than more traditional, in-person, venue-based recruitment. Previous research showed that venue-based recruitment can be successful [14,37,38], although many components factor into the success. Ott et al [39] explained that recruiting through local AIDS service organizations and testing centers may be a successful route for recruiting MSM engaging in risky sexual behaviors, although not all who are engaging in risky sexual behaviors will be engaged at AIDS service organizations. Although previous research has found engaging with AIDS service organizations to be a successful form of recruiting hardly reached populations, we found that engaging with local AIDS service organizations, community centers, shelters, youth groups, and churches did not offer any participants into our sample. Further research is warranted to explore the factors that lead to the very different levels of research engagement among young, substance-using, sexual and gender minority communities approached in virtual and physical spaces. It is possible that stigma and fear of being identified as substance-using may be a deterrent for in-person enrollment, especially if they are required to report their substance use or sexual behavior face to face to an in-person recruiter, compared with the anonymity that is offered online [6]. Additionally, individuals attending these venues, which included AIDS service organizations, shelters, youth groups, churches, bars, clubs, and community centers, could have had other purposes for attending, where screening for research was not a priority.

Facebook, Grindr, and Instagram yielded the largest number of eligible participants for the study from paid advertisements, while unpaid social media and dating applications yielded smaller enrollment numbers. The health research recruitment website, financially supported by the university, had a high recruitment rate, perhaps as people who received notifications from the portal had already indicated an interest in research participation and felt the project to be more legitimate if coming from a university-based email. Venue-based recruitment, however, yielded low numbers of participants recruited into the study (23/580, 3.9%), despite significant personnel and financial resources attributed to in-person recruitment. The enrollment rates for venue-based recruitment were consistently lower compared with paid social media advertising. Similar racial distributions were identified among online recruitment methods, but most participants who were recruited from venue-based sampling were White men, demonstrating that social media was more successful in terms of participant volume and racial diversity.

It was challenging to rapidly build relationships with venues, and we were denied from recruiting in some locations. Hiring and retaining Community Outreach Specialists proved to be more difficult than expected, with scheduling and driving complications being the most common issues. The distance for some outreach events, bars, and clubs extended up to 60 miles one way, and shifts were often in the late evening or night hours when venues were busiest. Rental cars and reimbursement for travel were expensive and time-consuming, which posed difficulties for active venue-based recruitment and maintaining Community Outreach Specialists throughout the recruitment time frame. We did not record data on recruiters’ perceptions of the barriers to venue-based recruitment.

There are several limitations in this paper; first, this analysis may not be generalizable to all young, substance-using, sexual and gender minority communities, as this sample included those based in southeastern Michigan only, and the analysis should be considered as a case study of recruitment for a specific trial in a specific context. Second, these responses may be subject to recall error, as all answers to the pretest survey were self-reported, and participants in the pretest survey may have suffered from desirability bias. Third, the length of the eligibility screener (~10 minutes to complete) was a limitation of venue-based recruitment, as potentially eligible participants often did not want to spend time completing the survey. Fourth, when tailoring advertisements, we focused imagery and keywords for those identifying as a man or male, which may have missed a large group of transgender and gender-diverse individuals. In the future, programs should offer a shorter eligibility screener to be taken on-site or a way to sign up to be contacted to take an eligibility screener. Additionally, tailoring verbiage to be more inclusive of gender-diverse and transgender communities is warranted. This analysis offers future researchers’ insight into recruiting into HIV- and STI-focused programs in southeastern Michigan.

Ultimately, web-based advertisements allow for potential participants to screen for eligibility at their convenience and are more convenient for programs looking to recruit young, substance-using, sexual and gender minority communities in southeastern Michigan. Generating a substantial sample of young, substance-using, sexual and gender minority communities in southeastern Michigan requires advertisements to be placed on multiple platforms, where resources are concentrated on web-based platforms such as Facebook, Instagram, and Grindr. Additionally, recruiting online offers a more cost-effective way to reach large numbers of people [22,23,29], including those who are hardly reached in southeastern Michigan. However, not everyone has access to a smartphone, computer, or internet, and future research efforts should consider this when designing recruitment efforts and screening surveys. Although online-based recruitment has shown to be cost-effective for some programs, we found it to be quite expensive. For example, recruiting from Snapchat cost over US $5000 per enrolled participant. Future programs aiming to recruit participants in southeastern Michigan should focus on paid, online advertisements, specifically Facebook, Instagram, and Grindr, rather than venue-based sampling to recruit hardly reached populations and young, substance-using sexual and gender minority communities.

## Abbreviations

AYAs: adolescents and young adults
CAPTCHA: Completely Automated Public Turing test to tell Computers and Humans Apart
GSA: Gay Straight Alliance
LGBTQ+: lesbian, gay, bisexual, transgender, queer, plus
MSM: men who have sex with men
STI: sexually transmitted infection
YMSM: young men who have sex with men
